# Supplementary material for: Do Nutrients and Nutraceuticals Play a Role in Diabetic Retinopathy? A Systematic Review
Source: Nutrients. 2022 Oct 21;14(20):4430. doi: 10.3390/nu14204430 (PMC9612209; doi:10.3390/nu14204430)
Supplement: Supplementary file 1 [file nutrients-14-04430-s001.zip › Supplementary Table S2.pdf]

Supplementary Table S2. Differences in gender, age and diabetes duration in diabetic subjects with and without retinopathy in the observational studies

	Gender (M/F or M%)		Age (years)		Diabetes duration (years)	
	DR	NDR	DR	NDR	DR	NDR
Zhao W. J., 2021 [50]	N=120/89	N=450/329	61 (55-68)	59 (53-67)	12 (6.7-17)	7 (2-12)
Gungor A., 2015 [44]	M=61%	M=50%	60.1±6.8	59.0.±5.1	NA	NA
Senyigit A., 2019 [71]	NA	NA	57.5±7.6	54.5±9.7	NA	NA
Herrmann M., 2015 [45]	NA	NA	NA	NA	NA	NA
Bajaj S., 2014 [46]	NA	NA	NA	NA	NA	NA
Ahmadieh H., 2013 [47]	NA	NA	NA	NA	NA	NA
Kaur H., 2011 [48]	NA	NA	NA	NA	NA	NA
Butler A.E., 2020 [42]	NA	NA	NA	NA	NA	NA
Ahmed L.H.M., 2020 [41]	NA	NA	57.4±9.9	54.0±9.7	19.2±9.3	10.7±7.9
Ahmed L.H.M., 2020 [49]	NA	NA	NA	NA	NA	NA
Millen A.E., 2003 [52]	NA	NA	NA	NA	NA	NA
Millen A.E., 2004 [51]	NA	NA	NA	NA	NA	NA
She C., 2021 [53]	40.3%	37.5%	63.2±8.5	65.4±8.8	13.1±8.5	8.6±5.7
Fahmy R., 2021 [55]	NA	NA	52.4±6.3	46.8±6.1	12.6± 8.1	7.5 ± 8.1
Cinici E., 2020 [60]	30/30	10/10	62.0±6.7	62.0±6.7	NPDR: 13.1<br>PDR: 18.3	6.0

Horikawa C., 2020 [62]	NA	NA	NA	NA	NA	NA
Malaguarnera G., 2015 [63]	75/70	55/41	NPDR: 64.1±10.8 PDR: 65.8±10.4	56.8±10.2	NA	NA
Satyanarayana A., 2011 [61]	M=55%	M=56%	55.7±8.2	54.7±9.3	11.0±6.9	10.2±6.9
Srivastav K., 2016 [59]	NPDR: N=13/7 PDR: N=10/10	N=12/8	NPDR: 54.3±6.9 PDR: 51.0±7.2	56.0±6.7	NPDR: 11.7±6.8 PDR: 11.7±6.4	6.0±7.0
de Luis D.A., 2005 [64]	NA	NA	NA	NA	NA	NA
Zhou Q., 2018 [54]	NA	NA	NA	NA	NA	NA
Yildirim Z., 2007 [56]	NPDR: N=12/13 PDR: N=8/17	N=12/13	NPDR: 63.5±9.0 PDR: 59.0±8.0	67.8±10.0	NA	NA
Ba-Ali S., 2018 [66]	N=14/11	N=19/10	61.6±9.1	63.1 ± 8.3	29.0±9.3	29.2 ±10.0
Hikichi T., 2011 [65]	NPDR: N=5/11 PDR: N=6/8	N=10/16	NPDR: 72.0±8.0 PDR: 56.0±12.0	66.0±11.0	NPDR: 14.0±7.0 PDR: 10.0±6.0	NA
Poorabbas A., 2007 [67]	NA	NA	NA	NA	NA	NA
Hattenbach L.O., 2000 [57]	NA	NA	NA	NA	NA	NA
Pękala-Wojciechowska A., 2018 [58]	NA	NA	NA	NA	NA	NA

Data are presented as mean ± SD or median and interquartile range.
Abbreviations: M, male; F, female; DR, Diabetic Retinopathy; NDR, None Diabetic Retinopathy PDR, Proliferative Diabetic Retinopathy; NPDR, Non Proliferative Diabetic Retinopathy; NA, not available
